# Anticancer Drug-Induced Cardiotoxicity: Insights and Pharmacogenetics

**DOI:** 10.3390/ph14100970

**Published:** 2021-09-25

**Authors:** Archana Adhikari, Syed Mohammed Basheeruddin Asdaq, Maitham A. Al Hawaj, Manodeep Chakraborty, Gayatri Thapa, Nihar Ranjan Bhuyan, Mohd. Imran, Mohammed Kanan Alshammari, Mohammed M. Alshehri, Aishah Ali Harshan, Abeer Alanazi, Bushra Dhuhayyan Alhazmi, Nagaraja Sreeharsha

**Affiliations:** 1 Pharmacology Department, Himalayan Pharmacy Institute Majhitar, Rangpo 737136, Sikkim, India; archanaadhikari6.aa@gmail.com (A.A.); thapagayatri95@gmail.com (G.T.); 2 Department of Pharmacy Practice, College of Pharmacy, AlMaarefa University, Dariyah, Riyadh 13713, Saudi Arabia; 3 Department of Pharmacy Practice, College of Clinical Pharmacy, King Faisal University, Hofuf 31982, Saudi Arabia; hawaj@kfu.edu.sa; 4 Department of Pharmaceutical Analysis, Himalayan Pharmacy Institute, Majhitar, Rangpo 737136, Sikkim, India; nihar.bhuyan@yahoo.com; 5 Department of Pharmaceutical Chemistry, Faculty of Pharmacy, Northern Border University, Rafha 91911, Saudi Arabia; imran.pchem@gmail.com; 6 Department of Pharmaceutical Care, Rafha Central Hospital, North Zone, Rafha 91911, Saudi Arabia; ii_kanan101@outlook.com; 7 Pharmaceutical Care Department, Ministry of National Guard-Health Affairs, Riyadh 11426, Saudi Arabia; M.Mansouralshehri@outlook.sa; 8 Department of Pharmaceutical Care, Northern Area Armed Forces Hospital, King Khalid Military City Hospital, Hafr Al-Batin 39745, Saudi Arabia; Ais7ah.har@gmail.com; 9 Department of Pharmaceutical Care, First Health Cluster in Eastern Province, King Fahad Specialist Hospital, Dammam 32253, Saudi Arabia; aalanaziabeer@gmail.com; 10 Faculty of Pharmacy, Northern Border University, Rafha 91911, Saudi Arabia; bushraalhaz96@gmail.com; 11 Department of Pharmaceutical Sciences, College of Clinical Pharmacy, King Faisal University, Al-Ahsa-31982, Saudi Arabia; sharsha@kfu.edu.sa; 12 Department of Pharmaceutics, Vidya Siri College of Pharmacy, Off Sarjapura Road, Bengaluru 560035, Karnataka, India

**Keywords:** anticancer drugs, cardiotoxicity, pharmacogenetics, radiation therapy, chemotherapeutic agent

## Abstract

The advancement in therapy has provided a dramatic improvement in the rate of recovery among cancer patients. However, this improved survival is also associated with enhanced risks for cardiovascular manifestations, including hypertension, arrhythmias, and heart failure. The cardiotoxicity induced by chemotherapy is a life-threatening consequence that restricts the use of several chemotherapy drugs in clinical practice. This article addresses the prevalence of cardiotoxicity mediated by commonly used chemotherapeutic and immunotherapeutic agents. The role of susceptible genes and radiation therapy in the occurrence of cardiotoxicity is also reviewed. This review also emphasizes the protective role of antioxidants and future perspectives in anticancer drug-induced cardiotoxicities.

## 1. Introduction

The advancement of medical science is associated with a paradigm shift in new anticancer therapies, causing a significant increase in long life expectancy in patients. Though a tremendous improvement has happened in cancer chemotherapy, the serious adverse effects associated with the therapy are still a major challenge [1,2,3]. Earlier studies reported a 0.5% prevalence rate of cancer in the general population with a mortality rate of 25%. The cytotoxic effects of the therapy affect all major organs and the clinical manifestations are associated with the development of co-morbidities [4]. Since cardiotoxicity is the most common adverse effect manifested by anticancer drug therapy, the increase in life expectancy owing to anticancer therapy may be negated by the enhanced death rate due to heart issues [5,6]. Cardiotoxicity can strike at any point during pharmacological treatment, with symptoms ranging from modest myocardial dysfunction to permanent heart failure and death [6].

The principle involved in chemotherapy is that it impairs the metabolic and mitotic processes of cancer cells and, in maximum cases, it also damages normal cells and tissues, which leads to various side effects ranging from mild to severe forms of gastrointestinal upset, the suppression of bone marrow, in addition to cardiovascular toxicities including myocardial dysfunction, heart failure, hypertension, and tachyarrhythmia [2,7].

Cancer therapies, including molecular target therapies, cytotoxic chemotherapy, and mediastinal irradiation, are seen to be linked with myocyte damage, ischemia, conduction and rhythm disturbances, left ventricular dysfunction, cardiac failure, and several other cardiovascular complications [3,8,9].

Various biochemical investigations into the different pathways of cardiovascular damage have been documented. Chemotherapeutic drugs cause cardiotoxicity by enhancing the formation of reactive oxygen and nitrogen species (ROS and RON), which impairs redox equilibrium. Although peroxisomes and other subcellular components are critical regulators of redox equilibrium, mitochondria remain the prime targets for anticancer-induced cardiotoxicity [10].

Anthracyclines (ANT) (doxorubicin), alkylating drugs (cyclophosphamide, cisplatin), and taxanes (paclitaxel, docetaxel) are the most common chemotherapeutic medications linked to serious cardiac events. More than half of the currently used anticancer therapies include anthracyclines, which cover breast cancer, sarcoma, gynecological cancer, and lymphoma [11]. 

This review aims to bring attention to several regularly used cancer medicines and their links to cardiotoxicity. Since each chemotherapeutic agent has a particular effect on the heart, this study covers the cardiac toxicities associated with different chemotherapeutic agents.

## 2. Mode of Cardiotoxicity Induction

### 2.1. Cyclophosphamide-Induced Carditoxicity

**Figure 1:**
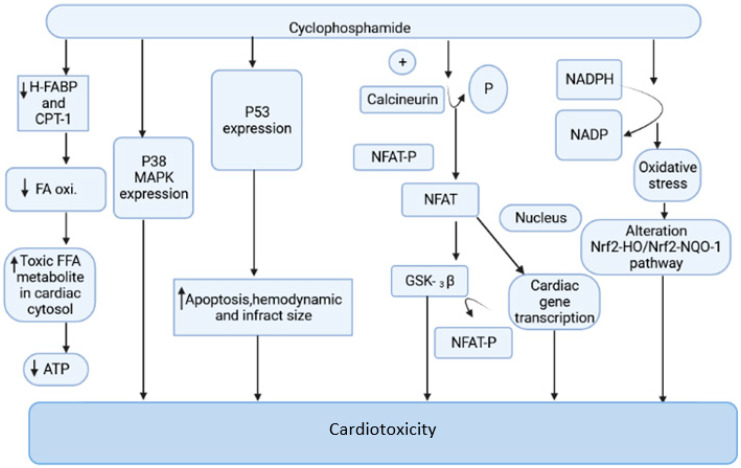
The mechanism of cyclophosphamide-mediated cardiac toxicity.

Cyclophosphamide is an anticancer drug with antitumor properties that is commonly used in humans for a range of neoplasms [12]. However, multiple reports have indicated that, in addition to having tumor-selective properties, cyclophosphamide has a slew of highly hazardous adverse manifestations (Figure 1), the most serious of which is cardiotoxicity [13].

Cyclophosphamide is activated by the cytochrome P-450 (CYP) enzyme in the liver, which transforms it into 4-hydroxycyclophosphamide. Aldo cyclophosphamide (AldoCY) and 4-hydroxycyclophosphamide are in equilibrium. AldoCY may be oxidized by aldehyde dehydrogenase 1 (ALDH1) to the inactive metabolite *o*-carboxymethylphosphoramide mustard (CEPM) or beta eliminated through a chemical mechanism that decomposes to produce cytotoxic phosphoramide mustard (PM) and the byproduct acrolein, depending on the cell type [14]. The antineoplastic activity of cyclophosphamide is due to the presence of phosphoramide mustard, which is the therapeutic metabolite in the active form present in cyclophosphamide, and it shows DNA alkylation activity, whereas acrolein, the other metabolite of cyclophosphamide, has the ability to interfere with the antioxidant system, further causing the generation of strongly active oxygen-free radicals, superoxide radicals, and hydrogen peroxide [15]. These free radicals are implicated in several enzyme inhibitions, lipid peroxidation, as well as membrane injury [16]. The generation of free radicals mediates oxidative stress associated with cyclophosphamide treatment witnessed by the following pathways. 

#### 2.1.1. Mitochondrial-Dependent ROS Production

Cyclophosphamide (CP) treatment is responsible for the generation of a tremendous amount of ROS, which damages the inner membrane of the mitochondria, resulting in a reduced capacity of the myocardial cell mitochondria to neutralize the toxic effect of ROS. The toxic effect of CP treatment is associated with a reduction in oxidative phosphorylation in mitochondrial cristae, which causes a decrease in ATP production and contributes to further damage in mitochondria [17,18,19]. This is one of the prime reasons for altered cardiac physiology and contractility. The increased expression of the pro-apoptotic molecule BAX in the mitochondrial membrane by CP treatment is responsible for the induction of apoptosis. The cleavage of caspases, inhibition of protein kinase, and the activation of phosphatase and increased intracellular PH are responsible for the stimulation of BAX proteins. The ratio of proapoptotic and antiapoptotic molecules Bax and Bcl2 makes the mitochondrial pathway susceptible to apoptosis. The disturbance in calcium homeostasis is also responsible for the initiation of apoptotic cell death [20,21]. The interaction of ROS and calcium is bidirectional. The calcium ions increase the production of mitochondrial ROS by stimulating respiratory chain activity. The released ROS acts on the endoplasmic reticulum to generate more calcium and ROS. This leads to the opening of the mitochondrial permeability transition pore, resulting in the release of pro-apoptotic factors [22,23].

#### 2.1.2. Oxidative Stress Produced by NADPH

CP-intoxicated cardiomyocytes are responsible for increased synthesis of NADPH oxidase, NADH dehydrogenase, and NADPH oxidase. The activation of these enzymatic systems is associated with the generation of free radical-mediated oxidative stress [24,25]. The myocardial cell when exposed to CP treatment increases NADPH oxidase and other mediators of ROS. The oxidative stress generated by the NADPH-mediated pathway is responsible for the alteration of the Nrf2-HO/Nrf2-NQO-1 pathway, which causes damage in the myocardial cell [16,25,26]. 

#### 2.1.3. Oxidative Stress and Nrf2 Expression

The leucine zipper protein Nuclear factor erythroid-2 related factor 2 (Nrf2) plays an important role in antioxidant regulation. Nrf2 is a major transcription factor that regulates the expression of genes involved in antioxidant and detoxifying enzymes [27,28]. Nrf2 protects against a number of pathological conditions associated with oxidative stress. It has been reported that Nrf2 can activate the autophagy-mediated clearance of different toxic protein aggregates generated due to the secondary effect of ROS formation. The myocardial cell exposed to CP is responsible for a decrease in Nrf2 expression responsible for DNA damage, while the excessive release of inflammatory cytokine levels is responsible for inflammation and death of myocardial cells [13].

#### 2.1.4. Endoplasmic Reticulum Stress Associated with CP

Oxidative stress and damage of mitochondria associated with CP administration generates a tremendous amount of stress in the myocardial endoplasmic reticulum. The toxic metabolite of CP acrolein contributes to the generation of ER stress, apoptosis, and the alteration of calcium homeostasis [21]. The imbalance of calcium homeostasis is mainly due to alterations in calsequestrin and the enzyme Ca++ ATPase (SERCA2a) present in the sarcoplasmic reticulum. As discussed in the previous sections, CP treatment is associated with the generation of ROS and mitochondrial damage, which may be a prime reason for the development of excessive stress conditions in the endoplasmic reticulum of myocardial cells. When these two pathological conditions coexist, they result in altered systolic and diastolic functions [29,30].

#### 2.1.5. Cyclophosphamide and Nitric Oxide

Different studies have reported the myocardial toxicity of nitric oxide (NO) generated by several anticancer drugs. CP exposure in the myocardial cell is responsible for an increase in the formation of NO due to the activity of eNOS and iNOS. On myocardial cells, eNOS and iNOS have been shown to have both protective and toxic effects [31,32]. The protective effect is due to the formation of NO, but generated NO when reacting with oxygen free radicals generates peroxynitrite (ONOO^−^), which is responsible for the toxic effects on the myocardial cell. It has been observed that iNOS is always associated with cardiotoxic events, but the effects of eNOS depend on the concentration of O_2_^−^. The exposure of CP treatment is responsible for the generation of NO and iNOS and causes the generation of nitrative stress. The generation of nitrative stress causes the induction of apoptosis by the activation of the p38/JNK cascade pathway [33,34,35].

### 2.2. Doxorubicin-Induced Cardiotoxicity

Doxorubicin (DOX) is an anthracycline antibiotic that was first derived from a bacterium called *Streptomyces peucetius* in the early 1960s and was first used as a cytotoxic drug in 1969 [36,37]. It is a highly successful chemotherapeutic medication for solid tumors, soft-tissue sarcoma, breast cancer, Hodgkin’s disease, Kaposi’s sarcoma, acute lymphoblastic leukemia, pediatric leukemia, lung cancer, lymphomas, and various metastatic malignancies [2,38,39]. The drug’s utility is limited owing to its large number of side effects, such as the suppression of the hematopoietic system, gastrointestinal disturbances, and baldness, with cardiac damage being the most dangerous [40,41,42].

The advanced conditions of cancer patients treated with repeated doses of doxorubicin for more than a month developed severe symptoms of myocardial toxicity with a prevalent rate of more than 30%. The wide spread of symptoms ranged from ventricular failure, a decrease in QRS segment, cardiac dilatation, tachycardia (150 beats/min), and hypotension (blood pressure 70/50 mmHg) [43]. The patients were unresponsive to inotropic drugs and mechanical circulatory assist devices. Several biomarker levels such as creatine phosphokinase, serum glutamic-oxaloacetic transaminase, and lactate dehydrogenase were also elevated. The histopathological examination reported decreased myofibrils, altered structure of the sarcoplasmic reticulum, vacuolization of the cytoplasm, mitochondrial inflammation, and elevated numbers of lysosomes [44,45]. Different experimental animals such as rats, mice, and rabbits treated with doxorubicin also reported similar symptoms of myocardial toxicities. The experimental animals in several studies in a reproducible manner showed the symptoms of cardiomyopathy and heart failure with the exposure of doxorubicin [46,47].

Multiple processes are involved in doxorubicin-induced cardiotoxicity, which has been linked to increased myocardial damage due to oxidative free radicals, as well as lower levels of sulfhydryl groups and antioxidants. In addition to myofibrillar deterioration and intracellular calcium dysregulation, doxorubicin-induced cardiac toxicity is known to cause myofibrillar deterioration [48,49]. Mitochondrial biogenesis is also hypothesized to be actively involved in doxorubicin-mediated cardiac damage due to its potential to stimulate the cell death pathway while blocking topoisomerase 2β [50,51]. Alterations in gene expression of the cardiac system, such as the expression of muscle-specific genes (cardiac actin, myosin light chain, and muscle creatine kinase), are shown to decrease in response to doxorubicin exposure in acute doxorubicin cardiotoxicity [52,53]. Endothelial dysfunction, an activated ubiquitin protease system, autophagy, and cell death collectively participate in doxorubicin-induced cardiotoxicity, as do NO release, impaired adenosine triphosphate (ATP) levels, iron regulatory protein (IRP) production, and augmented inflammatory mediator release [54,55]. 

#### 2.2.1. Mechanism of Doxorubicin-Induced Cardiotoxicity

##### Oxidative Stress

The generation of a tremendous amount of oxidative stress associated with DOX is the main culprit for the degeneration of myocardial cells. The imbalance between reactive oxygen species, reactive nitrogen species, and the intrinsic antioxidant systems is responsible for the development of oxidative stress. The following are some of the important cellular mechanisms that are responsible for the development of this oxidative stress [56,57].

A. Altered mitochondrial functions

The mitochondria fulfill the huge oxygen demand of cardiomyocytes. DOX treatment is responsible for structural changes in the mitochondria responsible for the depletion of energy production in the form of ATP [58,59].

The inner membrane of mitochondria contains an important component—cardiolipin. The interaction between cardiolipin and DOX is one of the major events responsible for cardiotoxicity associated with DOX. DOX and cardiolipin bind irreversibly due to the cationic charge of DOX and the anionic charge of cardiolipin, resulting in the accumulation of DOX in mitochondria. Cardiolipin has a major role in electron transport, but due to the formation of a complex with DOX, the activation of several enzymes is inhibited, resulting in an altered electron transport chain. Apart from this, DOX-mediated oxidative phosphorylation also plays an important role in the events of myocardial toxicity [60,61].

B. Fe–Dox complex

The hydroxy and ketone groups of DOX interact with Ferric ion (Fe3+) and form a complex. This complex interacts with the cell membrane and causes lipid peroxidation and generates free radicals. DOX is responsible for the accumulation of iron in mitochondria and initiates the event of apoptosis in the myocardial cell. DOX treatment is responsible for the inactivation of iron regulatory protein IRP1, and IRP2 and iron are transported into mitochondria by transport protein Mitoferrin-2. DOX causes the alteration of the post-translational modification of IRP1 and the recognition of iron-responsive elements is lost and results in altered iron homeostasis [62,63].

C. Role of NADPH in the generation of ROS

The catalytic activities of the enzymes nicotinamide adenosine dinucleotide phosphate (NADPH) and mitochondrial NADH dehydrogenase are responsible for the generation of free radicals. Angiotensin II is responsible for the elevation of NADPH oxidase and having a pivotal role in the generation of free radicals. DOX treatment is associated with the incremental genesis of nitric oxide synthase; nitric oxide reductase and P450 reductase enzymes contribute to the development of oxidative stress in the myocardial cell due to the formation of reactive oxygen species [64,65].

D. Generation of reactive oxygen species by nitric oxide

DOX treatment is responsible for the increased synthesis of nitric oxide mediated by the enzymes neuronal NO synthase, inducible NO synthase, and endothelial NO synthase. Nitric oxide is found in increased levels in damaged myocardial tissues. Nitric oxide by lipid peroxidation from peroxynitrite is responsible for the development of oxidative stress in mitochondria, resulting in necrosis and apoptosis [66,67].

E. Generation of oxidative stress by nrf2

The depletion of Nrf2 protein in DOX treatment is responsible for associated myocardial toxicities. The expression of Nrf2 is responsible for the induction of autophagy and maintains homeostasis between autophagy and oxidative stress [68].

##### Apoptosis

Through both the intrinsic and extrinsic pathways, DOX stimulates the apoptosis of the cardiac muscle cells. DOX toxicity results in an imbalance in various factors including an increase in oxidative stress, which activates HSF-1 (heat shock factor 1), thereby inducing HSP-25 (heat shock protein), and subsequently, p53 is equalized, leading to the production of proapoptotic factors such as Fas, FasL, and c-Myc, which is responsible for cardiac muscle cell death [69,70]. Another factor responsible for the cardiotoxicity associated with DOX is DOX-induced cardiotoxicity leading to the depletion of transcriptional factor GATA-4, which is responsible for regulating the apoptotic pathway via activating the anti-apoptotic gene Bcl-XL. In addition, DOX-induced cardiotoxicity is also found to increase active glycogen synthase kinase 3β (GSK3β), which is a negative regulator of GATA-4 in the nucleus [71]. Similarly, a role of TLR-2 (Toll-like receptor-2)-mediated cytokine production, cardiac dysfunction, and apoptosis by the activation of the pro-inflammatory nuclear factor-κB (NF-κB) pathway has been observed to be involved in DOX-induced cardiotoxicity [39]. Some studies showed that DOX-treated murine hearts inhibit Protein Kinase B (PKB/AKT), which is involved in the regulation of cell survival, proliferation, and metabolism. AKT is also found to play a critical role in decreasing oxidative stress via deactivating (GSK3β), which thereby decreases FYN nuclear translocation-mediated NF-E2-related factor 2 (Nrf2) nuclear export and degradation [72,73].

##### Necrosis

The various factors involved in the cellular necrosis in cardiomyocytes have typically been associated with cytoplasm and mitochondrial swelling, plasma membrane rupture, and coagulated sarcomere. Mitochondrial function disruption could have been attributed due to the dysregulation of lipid metabolism, the increased mitochondrial calcium level, and promoted mPTP opening that contributes to mitochondrial swelling and reduction in used ATP, and thus necrotic cell death is induced. DOX-induced cardiotoxicity has also been seen to be accompanied by disarray and a loss of myofilaments of the sarcomere since it is capable of degrading titin (a component of cardiac sarcomere) through the activation of the proteolytic pathway [74,75].

##### Pyroptosis

A new form of programmed cell death known as pyroptosis, which is characterized by cell lysis, cell swelling, and large bubbles blowing from the plasma, which further results in the release of cell contents and pro-inflammatory molecules, has been found to have been involved in the DOX-induced cardiotoxicity regulated by the Bnip3–caspase-3–GSDME pathway [76]. Pyroptosis can usually result in increased inflammation and can cause the activation of various caspases (caspase-1, caspase-3, caspase-4, and caspase-11 ), and it is also associated with the activation of NLR family pyrin domain containing 3 (NLRP3), leading to the cleavage of Gasdermin D (GSDMD) or GSDME and resulting in the rupture of the plasma membrane, which allows the release of interleukin-1beta (IL-1β) and IL-18, contributing to cardiac cell damage [77,78].

##### Autophagy

DOX treatment has a pivotal role in autophagy. DOX treatment has witnessed both the induction and inhibition of autophagy reactions. The reduction in the autophagy reaction is mediated by unc-51-like kinase 1 and AMPK pathways [79,80]. DOX is also associated with a decrease in GATA4 and Bcl-2 gene expression, whereas a rise in S6 kinase beta-1 expression can be observed, leading to an increase in autophagy gene expression. DOX treatment is responsible for the up-regulation of Atg12, Atg4, Atg5, and Bad genes. DOX-induced cardiomyopathy is associated with the up-regulation of autophagy marker LC3B. The induction and inhibition of the process of autophagy play an important role in doxorubicin-induced cardiomyopathy [81,82].

##### Fibrosis

The pathological process, like fibrosis, is also very common in doxorubicin-induced cardiotoxicity. DOX treatment is responsible for the development of interstitial fibrosis and perivascular fibrosis [83,84]. It is evident that the cell culture and animal model effects of DOX treatment on MMP-1 and MMP-2 genes are associated with the fibrosis of myocardial tissues. DOX is responsible for the inhibition of collagen synthesis by inhibiting transcription and translation responsible for the destruction of myocardial cells. The modulating effects of DOX treatment on phosphor-SMAD3 and transforming growth factor-beta (TGF-β) are responsible for the activation of the fibrosis signal pathway [85,86,87].

### 2.3. Trastuzumab-Induced Cardiotoxicity

This is a monoclonal antibody that is employed for the treatment of breast cancer patients who concurrently have an elevated human epidermal growth factor receptor 2 (HER2, also known as ErbB-21) level [88]. While therapeutic therapy with trastuzumab is reported to reduce morbidity and mortality in breast cancer patients, there are serious cardiac adverse effects that need to be checked [89,90]. HER2 is an important target for breast cancer and, similarly, a prime target for trastuzumab [91]. However, some mutant mouse models have documented that there are also some important roles regulated by ErbB-2 genes, such as in postnatal cardiomyocyte function and development [92]. Similarly, evidence suggests that ErbB-2 genes are involved in the normal functioning of the myocardium, which includes the involvement of a number of key pathways (such as phosphoinositide 3-kinase, mitogen-activated protein kinase, and focal adhesion kinase) that are required for cardiomyocyte maintenance, and the inhibition of apoptosis. Therefore, the blockade of ErbB signaling by trastuzumab could have been a major reason for its ability to induce cardiotoxicity [93,94].

**Figure 2:**
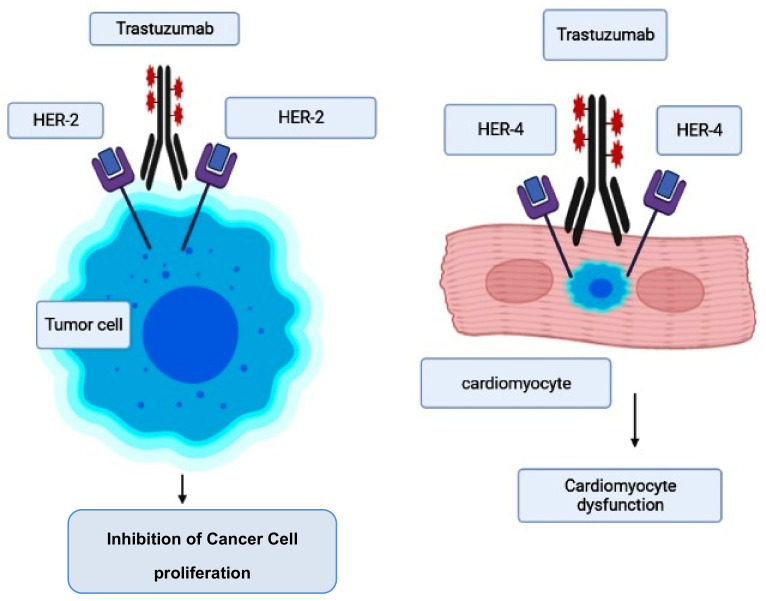
Mode of action of trastuzumab and its cardiotoxicity induction.

However, the risk of developing trastuzumab cardiotoxicity has been seen to increase in patients who receive concurrent anthracycline therapy [90,95,96]. When trastuzumab suppresses ErbB2 signaling, it was reported that it further accelerates the anthracycline’s ability to induce sarcomeric protein breakdown, increasing the chances of cardiac toxicity and heart failure [97]. The pathogenesis and molecular mechanism involved in trastuzumab-induced cardiotoxicity are given in Figure 2 [98].

### 2.4. Fluorouracil-Induced Cardiotoxicity

**Figure 3:**
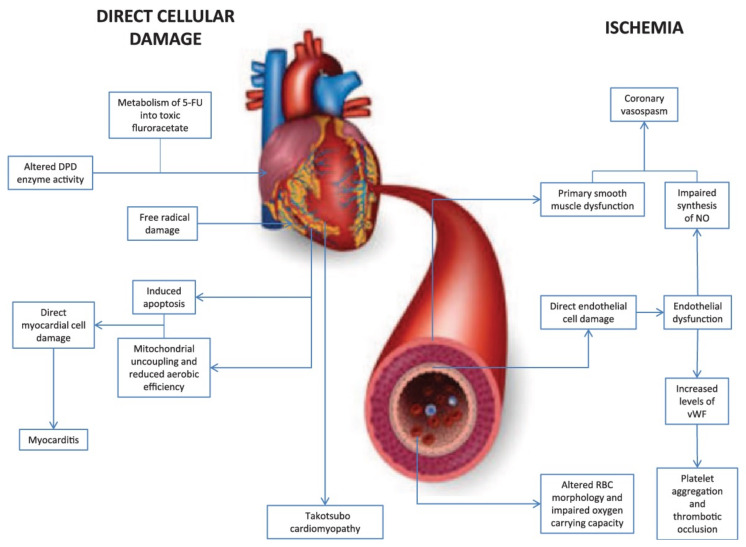
Diagrammatic representation of two major mechanisms of 5-fluorouracil-induced cardiotoxicity.

5-fluorouracil (5-FU) is a pyrimidine analogue chemotherapy medication employed in the treatment of a number of cancer types, including colorectal, breast, gastric, pancreatic, prostate, and bladder cancers, among others [99,100]. Myelosuppression, diarrhea, stomatitis, nausea, vomiting, and baldness are common side effects of the medication. Furthermore, 5-FU has been linked to cardiotoxicity, which includes myocardial infarction, cardiac arrhythmias, altered blood pressure, left ventricular failure, cardiac arrest, and sudden death [99]. The potential mechanisms involved in 5-FU-induced cardiotoxicity are given in Figure 3 [101]. Angina is noticeable mostly during infusions, and occasionally it is delayed until a few hours after 5-FU application. The incidence of angina associated with the use of 5-FU ranges from 1.2 to 18 percent [102,103,104,105]. Cardiotoxicity that is severe or life-threatening or ventricular arrhythmias, on the other hand, are far less common, with an incidence of about 0.55 percent [106]. When compared to anthracyclines, cardiac adverse effects of 5-FU are rare, with an incidence of 1.2–7.6%, and life-threatening cardiotoxicity of 5-FU has been documented in less than 1% of cases [107]. Though the exact processes of 5-FU-mediated cardiac toxicity are yet unknown, spasm of the coronary artery is suggested as a hypothesis [64]. Ultrasound and angiography have been used in several investigations to show that 5-FU infusion causes both coronary and brachial artery vasospasm. Patients with coronary vasospasm may have ECG findings suggestive of coronary occlusion, such as ST-segment elevation and biochemical changes in myocardial injury with increased troponin. As a result, it is recommended that when 5-FU is given to cancer patients, a high index of suspicion for probable cardiac toxicity be maintained [108].

### 2.5. Cisplatin-Induced Cardiotoxicity

Cisplatin, commonly known as cis-di ammine dichloroplatinum (CDDP), is a highly effective chemotherapy drug [109]. In diseases such as ovarian and cervical cancer and testicular cancer, it is used alone or in combination regimens [110]. However, due to adverse effects, including toxicities to the kidney, liver, and gastrointestinal disturbances, cisplatin’s clinical use is restricted. Despite these side effects, many survivors of cisplatin treatment may develop acute or chronic cardiovascular problems, which can negatively impact their quality of life [111]. CDDP-induced cardiotoxicity has been linked to ventricular and supraventricular arrhythmias, occasional sinus bradycardia, alterations in electrocardiography, occasional total atrioventricular block, and congestive heart failure. With cisplatin-based chemotherapy, oxidative stress is considered as a major reason for cardiac toxicity [112]. It has also been shown that cisplatin-based chemotherapy causes a decrease in the concentration of different antioxidants in patients. It may also cause ROS generation by accumulating in mitochondria [113]. However, it has been documented that all of these factors collectively lead to congestive heart failure and sudden cardiac death. Table 1 summarizes the adverse cardiotoxic consequences of chemotherapy [114].

### 2.6. Immunotherapy-Induced Cardiotoxicity

With cancer progression, initially, the body’s immune system prevents tumor outgrowth. However, cancer cells can escape the various pathways that provide immunologic antitumor responses, and such pathways include immune system inhibitory pathways such as cytotoxic T lymphocyte-associated antigen 4 (CTLA-4), programmed cell death ligand 1 (PD-L1), and programmed cell death 1 (PD-1) (natural checkpoints that dampen the antitumor responses of T cells). Therefore, various studies were carried out to determine if by stimulating an antitumor immune response in cancer patients, cancer could be fought better. Since then, immunotherapy came into existence, and this is divided into passive immunotherapies such as monoclonal antibodies, checkpoint inhibitors, cytokine therapy, bispecific T cell engager and antitumor vaccines or adoptive T cell transfer, which dramatically improved outcomes for wide varieties of cancer, among which adoptive T cell therapy and immune checkpoint inhibitors are most widely used and are used as longer duration therapy. However, having the benefit of activated immune response came with a price, as both the therapies have been reported to cause various cardiovascular complications such as hypotension, arrhythmia, left ventricular dysfunction, myocarditis, with clinical presentations ranging from asymptomatic cardiac biomarker elevation to heart failure, and cardiogenic shock [115,116,117,118,119].

#### 2.6.1. Immune Checkpoint Inhibitors and Cardiac Complications

Immune checkpoint inhibitors (ICIs) show their action by amplifying T cell-mediated immune response against cancer cells, by blocking the intrinsic down-regulators of immunity, such as programmed cell death 1 (PD-1), programmed cell death ligand 1 (PD-L1), and cytotoxic T-lymphocyte antigen 4 (CTLA-4). These immune checkpoint inhibitors can induce tumor responses in different types of tumors including non-small cell lung cancer, renal cancer, melanoma, and Hodgkin’s disease. Although ICIs have brought improvement in the treatment of many aggressive malignancies, several studies have reported that PD-1 deletion and CTLA-4 inhibition can cause serious cardiac complications. When an experiment was carried out in a mouse model, it was reported that with the loss of the PD-1 or CTLA-4 receptor, significant infiltration of CD4+ and CD8+ T cells took place, resulting in the development of a dilated cardiomyopathy. However, the exact mechanism underlying cardiac toxicity associated with checkpoint inhibitors has not been studied comprehensively, and immune-mediated myocarditis could also be the exaggerated adaptive immune response against shared epitopes in the myocardium and tumor cells [120,121,122,123,124].

#### 2.6.2. CDK4/6 Inhibitors

Cyclins have a major role to play in regulating the cell cycle. They show their activity by interacting with their partner serine/threonine CDKs (cyclin-dependent kinases). There are different types of CDKs available. CDKs 1–6 have a major role in coordinating cell cycle progression, whereas CDKs 7, 8, and 9 show downstream effects as transcriptional regulators. Among all the CDKs, the major target in cancer therapy is CDK 4/6, since it is required for the initiation and progression of various malignancies and is usually hyperactive in cancers [125,126]. CDKs generally help in regulating the cellular transition from G1 phase to S1 phase in the cell cycle, and the inhibitors (CDK 4/6 inhibitors) show their action by blocking the proliferation of cancer cells by effectively inducing G1 cell cycle arrest. However, CDK inhibitors have been seen to cause cardiac complications. One major concern about the use of CDK inhibitors is that they have been seen to potentially increase the QTc interval, which is most commonly seen with ribociclib. Some studies showed that ribociclib is capable of down-regulating the expression of KCNH2 (which encodes for the potassium channel Herg) and up-regulating the expression of SCN5A and SNTA1 (which encode for the sodium channels syntrophin-α1 and Nav1.5), which are the genes associated with long QT syndrome. There is also evidence that shows the increasing risk of thromboembolic events with the use of CDK inhibitors. However, there are limited data available regarding the cardiac safety of CDK 4/6 inhibitors. Therefore, it requires detailed investigation to understand the exact mechanism [127,128].

#### 2.6.3. VEGF Inhibitors

Anti-VEGF agents are novel drugs recently introduced in the treatment of different types of cancer. These drugs are responsible for the inhibition of angiogenesis induced by cancer tissues. VEGF is responsible for maintaining the integrity of blood vessels. Therefore, long-term systemic administration of anti-VEGF as anticancer therapy can cause several side effects. Cardiovascular side effects such as hypertension and the impairment of cardiac functions are well documented with the treatment of anti-VEGF drugs [129].

Patients who are under the therapy of bevacizumab reported the symptom of hypertension with a prevalence rate of 32%, whereas for sunitinib treatment it was 28% in the phase II clinical trial and 15% in the phase III clinical trial. Patients who are under the therapy of sorafenib report an incidence rate of 17% [130].

The prime mechanism behind the hypertension associated with anti-VEGF agents is the reduction in nitric oxide (NO) synthesis by the walls of arterioles. NO synthesis is mediated by the up-regulation of endothelial NO synthase, and anti-VEGF agents cause a decrease in NO synthesis. The decrease in NO synthesis results in vasoconstriction and promotes peripheral resistance resulting in hypertension [131].

Altered cardiac functions are also evident in patients under the treatment of anti-VEGF agents such as sunitinib or sorafenib. A total of 15% and 11% of patients who received sunitinib and sorafenib, respectively, for the treatment of renal cancer developed decreased left ventricular ejection. In a randomized phase III trial, 2.9% of patients under the therapy of sorafenib for the treatment of advanced renal cancer developed cardiac ischemic conditions. In an animal model, mice treated with the ectodomain of VEGFR-1 for 2 weeks reported a 32% decrease in cardiac output [132].

#### 2.6.4. Chimeric Antigen Receptor (CAR) T Cell Therapy

**Figure 4:**
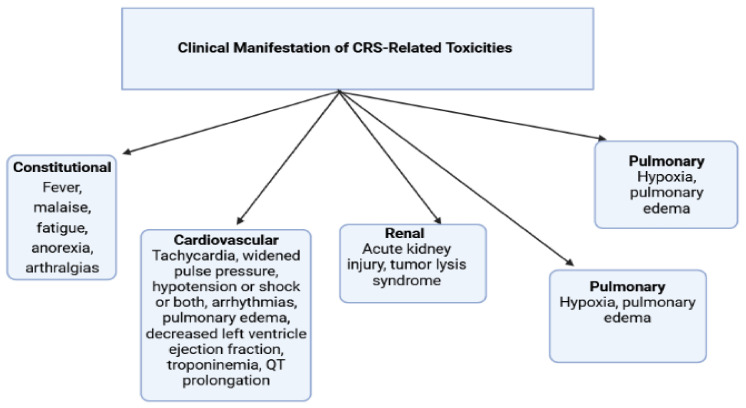
Clinical manifestation of CRS-related toxicities.

Although CAR T cell therapies have been reported to have robust clinical efficacy in hematological malignancies, similarly, their treatment-related comorbidities are increasingly becoming a measure of concern. CAR T cell therapy has usually been found to have been accompanied by toxicities, among which the most common one is CRS (cytokine release syndrome). CRS involves symptoms such as high fever, malaise, fatigue, and anorexia, and it has been seen to cause toxicities in various organs as well, such as the cardiovascular system, nervous system, respiratory system, gastrointestinal tract, hepatic system, hematological system, and the renal system, as shown in Figure 4. From all the other adverse effects associated with CAR T cell therapy, cardiovascular events were seen to occur in between 10 and 36% of the patients, ranging from arrhythmia, tachycardia, hypotension, and decreased left ventricular systolic function to cardiogenic shock and death. The various mechanisms associated with cardiovascular complications have been poorly understood, but they could be multifactorial. The pathophysiology of cardiac dysfunction with CRS resembles cardiomyopathy during stress and sepsis, likely associated with IL-6 (interleukin-6), which is usually found as a mediator of myocardial depression in cases of inflammatory and infectious states. However, the onset of cardiac dysfunction can be either acute or severe, but usually it is reversible [133,134,135].

## 3. Genes’ Susceptibility to Cardiotoxicity Induced by Chemotherapeutic Agents

Genes that are responsible for cardiotoxicity are found to have alleles that initiate the disease. Some of these genes are reactive oxygen species [90], nicotinamide adenine dinucleotide phosphate oxidase [136], P450 oxidoreductase, glutathione S-transferase, renin–angiotensin system-related genes, titin-truncating variants, histamine N-methyltransferase, DNA methyltransferase 1, G protein-coupled receptor 35, ultraviolet irradiation resistance-associated gene, general control nonderepressible 2, eukaryotic initiation factor 2, uncoupling protein 2, B-cell lymphoma-2, catalase, hyaluronan synthase 3, superoxide dismutase, T cell leukemia/lymphoma 1A, human leukocyte antigen, Toll-like receptor 2 and 9, heme oxygenase-1, carbonyl reductase, carbonyl reductase 1 and 3 [137,138], ERBB2 genes, CUGBA Elav-Like Family Member 4, Retionic Acid Receptor Gamma, Solute Carrier Family 28 Member 3, and UDP-Glucuronosyltransferase 1–6 [139].

### 3.1. ErbB2 Gene 

ErbB2, also known as HER2/Neu, is a membrane receptor that belongs to the ErbB1, ErbB3, and ErbB4 families of epidermal growth factor receptors. It is generally activated by interacting (dimerizing) with another ErbB receptor once that receptor is triggered by a ligand. To date, six monoclonal antibody-based therapeutics such as cetuximab, necitumumab, panitumumab, trastuzumab, pertuzumab, and ado-trastuzumabemtansine and five tyrosine kinase inhibitors (TKIs) such as erlotinib, gefitinib, lapatinib, vandetanib, and afatinib that target ErbB family members are recognized by the US FDA for clinical use [140]. ErbB2’s clinical link with the functioning of the heart was reported in a study where 27% of the patients who received doxorubicin and anti-ErbB2 (trastuzumab/herceptin) for breast cancer with overexpression of ErbB2 subsequently developed synergistic cardiac toxicity. According to certain studies, when trastuzumab is given after anthracyclines, the chances for cardiac toxicity are enhanced, resulting in a substantial decrease in left ventricular ejection fraction (LVEF) in around 25% of patients or symptomatic heart failure in 0.8–4.0 percent of patients [141]. When an experiment was conducted to better understand the effect of ErbB2 in adult heart mutant mice with a cardiac-restricted removal of ErbB2, these ErbB2 mutants showed a number of signs of dilated cardiomyopathy, including chamber dilation, wall thinning, and decreased contractility. Because these genes are also important in the maintenance of normal cardiac activity, treatment with trastuzumab and other monoclonal antibodies in a patient with overexpression of ErbB2 resulting in cardiac dysfunction has been linked to the suppression of the ErbB2 gene [142].

### 3.2. NOX2

The two forms of Nox enzymes, Nox2 and Nox4, are excessively shown in the mammalian heart. The activation of Nox2 is generally carried out by angiotensin II, endothelin-1, tumor necrosis factor-β, growth factors, and mechanical force. Several studies have reported Nox2-dependent ROS generation mediating congestive heart failure [143]. Although the antibiotic doxorubicin (DOX) is a commonly used and effective first-line cancer treatment, it can cause cardiotoxicity, which is linked to a rise in myocardial reactive oxygen species generation (ROS). Recent research suggests that ROS produced by Nox2 NADPH oxidase plays a role in essential pathways causing cardiac dysfunction caused by chronic DOX therapy [144,145]. Although the significance of Nox2 NADPH oxidase in cardiac damage caused by doxorubicin has not been confirmed to date, doxorubicin has been shown to enhance myocardial NADPH oxidase in vivo and to stimulate MMP-2 function and apoptosis in vitro through the NADPH oxidase-dependent activation of c-Jun NH2-terminal kinase (JNK)/extracellular signal-regulated kinase (ERK) and hydrogen peroxide. Further, doxorubicin increases the synthesis of various recognized triggers of NADPH oxidases, which are critical in cardiac remodeling [146,147].

### 3.3. CBR Gene

Carbonyl reductase (CBR) is an oxidoreductase protein that is a part of the short-chain dehydrogenase reductase (SDR) family [148]. The conversion of anthracyclines to secondary alcohol metabolites (i.e., doxorubicinol, daunorubicinol) catalyzed by CBR has been suggested as a possible mechanism of anthracycline cardiotoxicity [149,150]. The well-known cardiomyopathy linked to anthracycline (doxorubicin) treatment was examined in vitro and ex vivo, indicating that the C-13 hydroxy metabolite doxorubicinol may play a crucial role in the development of DOX-induced cardiotoxicity. When mice with overexpression of human CBR were used in an experiment, there was a high frequency of the development of cardiac toxicity when compared to normal animals [63]. According to certain studies, a single nucleotide polymorphism (SNP) in CRB can affect the enzyme’s pharmacokinetics and toxicity [151,152]. 

### 3.4. TTN Gene

The use of doxorubicin in breast cancer therapy was responsible for developing familial and sporadic dilated cardiomyopathy due to the altered TTN gene. Linschoten et al. in their study observed two women who suffered from heart failure after months of receiving anticancer therapy. The genetic screening study witnessed mutations in TTN, encoding the myofilament titin responsible for the occurrence of heart failure [153].

### 3.5. G Protein-Coupled Receptor 35 (GPR35)

The overexpression of GPR35 is responsible for a significant reduction in cell viability and altered morphological changes in cardiomyocytes. Ruiz-Pinto et al. observed the treatment of anthracyclines in 83 cancer patients and found a strong association between chronic ACT treatment and GPR35. This treatment protocol was associated with severe symptomatic cardiac presentation [154].

### 3.6. CELF4, RARG, SLC28A3, UGT1A6

Anthracycline treatment witnessed clinically significant cardiac toxicity due to a generic polymorphism in CELF4, RARG, SLC28A3, and UGT1A6 genes responsible for alterations in anatomical and physiological characteristics of the sarcomere, the expression of topoisomerase-2β, the transportation of drugs, and the biotransformation of drugs, respectively [155]. 

## 4. Radiation Therapy-Induced Cardiotoxicity

Cardiovascular harm can occur even when the heart is only partially exposed to radiation therapy. CHF, restrictive cardiomyopathy, coronary artery disease, valvular heart disease, constrictive pericarditis, and pericardial effusion are all conditions that affect the heart [156]. However, the chances of cardiac toxicity are found to be higher in patients undergoing radiation therapy along with the contemporary administration of chemotherapeutic agents [157]. Radiation therapy is used to treat diseases such as lymphoma, breast cancer, thymoma, high and low respiratory tracts, and esophageal and gastric lesions, among others. Radiation therapy-induced heart disease, on the other hand, was most typically detected after radiotherapy for malignant tumors in the chest, particularly left breast cancer [158]. Breast cancer is one of the most frequent cancers in women, impacting women all over the world. Radiation therapy is now considered as part of the standard care among the different therapies used to treat breast cancer as it is found to reduce mortality. However, long-term cardiotoxicity in breast cancer patients under radiation therapy has been linked to a range of 1.2 to 3.5 times greater chances of cardiac toxicity due to higher exposure to the heart. The mechanism underlying radiation therapy-induced cardiotoxicity is unknown, although it is thought to entail the development of inflammation, which leads to endothelial dysfunction, microvascular alterations, and the acceleration of the atherosclerotic process in coronary vessels. Various studies are working on preparing appropriate dose limitations for the heart and developing approaches that can reduce the potential cardiotoxic effect of radiation therapy [159].

## 5. Role of Herbs as Antioxidants in the Inhibition of Anticancer Drug-Induced Cardiac Toxicity

The utilization of herbs and herbal-based therapy against several disease conditions is gaining popularity throughout the world due to its potency and apparent safety profile. Traditional healers are using plant-based therapy from ancient times to deal with several clinical manifestations. The potent phytoconstituents present in different parts of plants have a strong antioxidant capacity. To deal with severe drug-induced toxicities such as anticancer drug-induced organ toxicities, natural antioxidants obtained from plant sources are found to be very beneficial (Table 2). Different preclinical studies reported significant protective activity against anticancer drug-induced organ toxicities [14].

**Table 2:** Role of medicinal herbs and their active constituents against cardiac toxicity due to anticancer drugs.

References	Animals Used	Method and Intervention	Major Findings
Bhatt et al. [12]	Wistar rats	Rats were exposed to CP toxicity with the dose of (200 mg/kg, i.p.) on day 1 of the treatment protocol. The animals were treated with 100 mg/kg of mangiferin for 10 days.	The treatment with mangiferin restored serum biomarker enzymes, antioxidant levels, lipid profile, electrocardiographic parameters, and histological score and mortality.
Ayza et al. [16]	Sprague Dawley rats	Rats were exposed to CP toxicity with the dose of (200 mg/kg, i.p.) on day 1 of the treatment protocol. The animals were treated with 100 mg/kg of mangiferin for 10 days.	The crude extract and ethyl acetate and aqueous fractions of Croton macrostachyus exhibited in vitro free radical scavenging activities in DPPH free radical scavenging assay. The treatment also restored serum biomarker enzymes, lipid profile, and histological score.
Qin et al. [32]	Wistar rats	Rats were exposed to CP toxicity with the dose of (07 mg/kg, i.p.) on day 6 of the treatment protocol. The animals were treated with 5, 15 and 45 mg/kg of resveratrol for 10 days.	Resveratrol treatment reported synergistic antineoplastic activity with cisplatin to A549 adenocarcinoma cells. Resveratrol treatment in a dose-dependent manner restored blood pressure, heart rate, serum biomarker enzymes, tissue antioxidant level, and histopathology of myocardial cell against CP-induced cardiotoxicity.
Bahadır et al. [103]	Wistar rats	Rats were exposed to cisplatin toxicity with the dose of 5 mg/kg/week for two weeks. The animals were treated with curcumin (200 mg/kg) and beta-carotene (100 mg/kg)	Curcumin and beta-carotene reported significant improvement in tissue antioxidant levels, tumor necrosis factor-α, interleukin-1β, and interleukin-6 against cisplatin-induced cardiotoxicity.
El-Hawwary et al. [106]	Wistar rats	Rats were exposed to cisplatin toxicity with the dose (2 mg/kg/day) for 1 week. The animals were treated with ginger (500 mg/kg) for 12 days.	Ginger treatment reported significant restoration of cardiac histology ultrastructure and a decrease in P53 and TNF-α immune expressions and creatinine kinase and lactate dehydrogenase levels against cisplatin-induced cardiotoxicity.
Ahmed et al. [157]	Wistar rats	Rats were exposed to cisplatin toxicity with the dose of doxorubicin (25 mg/kg i.p.) on 7th day. The animals were treated with methyl gallate (150 and 300 mg/kg) for 7 days.	Methyl gallate treatment restored ECG recording, serum biomarkers, and tissue antioxidant and lipid profile levels against doxorubicin-induced cardiotoxicity.
Birari et al. [158]	Wistar rats	Rats were intoxicated with a dose of (6 mg/kg, i.p.) with doxorubicin on alternate days (cumulative dose 30 mg/kg). The rats were treated with aloin as aqueous solution (1, 5, 25 and 125 mg/kg, p.o., once a day)	Aloin treatment restored ECG tracings and tissue antioxidant levels and reduced the levels of proinflammatory cytokines TNF-α, IL-1β, and IL-6 against doxorubicin-induced cardiotoxicity.
Hu X et al. [159]	C57BL/6 mice	Mice were administered with doxorubicin with a dose of (15 mg/kg, i.p.). Mice were treated with asiatic acid (10 mg/kg and 30 mg/kg) two weeks before doxorubicin treatment	Asiatic acid treatment restored echocardiographic and tissue antioxidant level. Asiatic acid reduced oxidative stress and apoptosis induced by doxorubicin by AKT signaling pathway.
Meng et al. [160]	C57BL/6 mice	Mice were administered with doxorubicin with a dose of (15 mg/kg, i.p.). Mice were treated with geniposide (25 mg/kg and 50 mg/kg) for 10 days, which was started three days before doxorubicin treatment.	Geniposide witnessed cardio-protection against doxorubicin-induced cardiotoxicity by the activation of AMP-activated protein kinase α.
Zhang et al. [161]	C57BL/6 mice	Mice were treated with doxorubicin with a dose of (20 mg/kg, i.p.). Mice were treated with oroxylin A for 10 days, which was started five days before doxorubicin treatment	Oroxylin A treatment restored oxidative damage and reduced inflammation accumulation and myocardial apoptosis in vivo and in vitro. Oroxylin A showed protection by activation of sirtuin 1 signaling pathway via the cAMP/protein kinase A.

## 6. Proposed Future Hopes

The wide availability of different potential phytoconstituents such as catechins, flavonoids, anthocyanin, flavones, isocatechins, and isoflavones means that they are potential candidates for relieving free radical stress mediated by oxygen free radicals [16].

Polyphenolic compounds have the capacity to inhibit vasoconstrictor endothelin-1 present in endothelial cells and inhibit angiogenesis in smooth muscle cells by the down-regulation of endothelial growth factor and matrix metalloproteinase-2 [162,163,164]. Polyphenols are also responsible for the reduced production of cytokines associated with inflammation and vascular adhesion [165].

Apart from antioxidant capacity associated with phenolic compounds and flavonoids, they also have potential action on different enzyme systems such as lipoxygenase, matrix metalloproteinases, cyclooxygenase, xanthine oxidase, angiotensin-converting enzyme, cytochrome P450, and proteasome, which have very important roles in signal transduction pathways. Flavonoids also have a role in drug transport at the cellular level. These targets can be utilized to develop strategies for anticancer drug-induced myocardial toxicities [166] (Table 3).

## 7. Conclusions

This article provides a comprehensive overview of the incidence of cardiotoxicity caused by commonly used chemotherapeutic and immunotherapeutic drugs, susceptible genes, and radiation therapy, and the protective role of antioxidants and other treatments as future expectations to combat anticancer cardiotoxicities. Cardiac toxicity is the most common adverse effect of practically all anticancer medications currently on the market, including anthracyclines, inhibitors of the epidermal growth factor receptor type 2 (anti-HER2), and antimetabolites. Apoptosis, autophagy, increased oxidative stress, and the inhibition of heart contractile function are among the mechanisms behind the negative effects on the cardiovascular system. Immunotherapy, which includes T cell therapy and immune checkpoint inhibitors, is also associated with a significant level of cardiotoxicities. Radiation therapy, responsible for augmenting inflammatory reactions, also contributes to the events of myocardial toxicity. The antioxidants obtained from herbal sources were found to be beneficial to curb anticancer drug-induced cardiotoxicities. Cardiotoxicity due to anticancer therapy needs to be kept in mind as an essential factor that can negate the positive outcomes of the anticancer regimen and may impact the prognosis and possible recovery of the patient. Indeed, this poses a challenge for health care professionals dealing with patients, particularly oncologists and cardiologists, and especially when new anticancer agents with significant anticancer potency fail to show a good safety index. Regular examination of patients for possible cardiac toxicity is an essential step to alleviating the development of later stage complications. Additionally, there is a need to conduct a thorough examination of each drug’s cardiotoxicity mechanisms, which would aid in the development of appropriate cardioprotective strategies.

## Figures and Tables

**Table 1:** Different types of cardiotoxic effects of chemotherapeutic agents.

Drugs Causing Ischemia or Thromboembolism	Cisplatin, Thalidomide, Fluorouracil, Capecitabine, Paclitaxel, Docetaxel, Trastuzumab, Anthracyclines/Anthraquinones, Cyclophosphamide
Drugs that Cause Hypertension	bevacizumab, cisplatin, sunitinib, sorafenib
Tamponade and Endomyocardial Fibrosis	busulfan
Autonomic Neuropathy	vincristine
Bradyarrhythmias	paclitaxel
Myocarditis with Hemorrhage (rare)	cyclophosphamide (high-dose therapy)
Pulmonary Fibrosis	bleomycin, methotrexate, busulfan, cyclophosphamide
Raynaud’s Phenomenon	vinblastine, bleomycin
Torsades de Pointes or QT Prolongation	arsenic trioxide

**Table 3:** Different agents under trial for anticancer drug-induced cardiac toxicity [166].

Title	Drugs under Trial	Phase	Justification of the Study
Anticancer Drug-Induced Cardiac Toxicity in High-Risk Patients (NCT00292526)	Enalapril	Phase 4	The patients who received chemotherapy experienced an elevation of troponin I responsible for development of left ventricular dysfunction and altered cardiovascular functions. The activation of renin–angiotensin system is responsible for development of several myocardial dysfunctions and resulted in chemotherapy-induced cardiotoxicity (CTIC). This study found the effect of ACE inhibitors in the prevention of CTIC in high-risk cancer patients.
Protective Effects of the Nutritional Supplement Sulforaphane on Doxorubicin-Associated Cardiac Dysfunction (NCT03934905)	Sulforaphane as supplement	Phase 2	Sulforaphane (SFN) is responsible for activation of transcription factor Nrf2 and induces defense mechanisms in normal cells. SFN was shown to inhibit carcinogenesis and metastases and increase the sensitivity of cancer cells to doxorubicin.
Cardiotoxicity Prevention in Breast Cancer Patients Treated with Anthracyclines and/or Trastuzumab (NCT02236806)	Bisoprolol and ramipril	Phase 3	This study showed a protective effect of beta blockers and ACE inhibitors against breast cancer patients treated with anthracyclines with or without trastuzumab.
STOP-CA (Statins TO Prevent the Cardiotoxicity from Anthracyclines) (NCT02943590)	Statins	Phase 2	This study demonstrated the protective effect of atorvastatin, a drug used in the treatment of hyperlipidemia against doxorubicin-induced cardiac damage.
Carvedilol Effect in Preventing Chemotherapy-Induced Cardiotoxicity (NCT01724450)	Carvedilol	Phase 3	This research found the preventive effect of carvedilol against chemotherapy-induced cardiotoxicity in breast cancer patients.
Prevention of Chemotherapy-Induced Cardiotoxicity in Children with Bone Tumors and Acute Myeloid Leukemia (NCT03389724)	Captopril	Phase 3	This study found the protective effect of captopril against chemotherapy-induced cardiotoxicity in children with bone tumors and acute myeloid leukemia.

## Data Availability

Data sharing not applicable.
